# Role of methionine adenosyltransferase 2A in bovine preimplantation development and its associated genomic regions

**DOI:** 10.1038/s41598-017-04003-1

**Published:** 2017-06-19

**Authors:** Shuntaro Ikeda, Ryouka Kawahara-Miki, Hisataka Iwata, Miki Sugimoto, Shinichi Kume

**Affiliations:** 1 Laboratory of Animal Physiology and Functional Anatomy, Graduate School of Agriculture, Kyoto University, Kyoto, 606-8502 Japan; 2 NODAI Genome Research Center, Tokyo University of Agriculture, Tokyo, 156-8502 Japan; 3 Department of Animal Sciences, Tokyo University of Agriculture, Kanagawa, 243-0034 Japan

## Abstract

Methionine adenosyltransferase (MAT) is involved in folate-mediated one-carbon metabolism, which is essential for preimplantation embryos in terms of both short-term periconceptional development and long-term phenotypic programming beyond the periconceptional period. Here, our immunofluorescence analysis of bovine oocytes and preimplantation embryos revealed the consistent expression of MAT2A (the catalytic subunit of the ubiquitously expressed-type of MAT isozyme) during this period. Addition of the MAT2A inhibitor FIDAS to the culture media of bovine preimplantation embryos reduced their blastocyst development, revealing the particular importance of MAT2A in successful blastocyst development. Exploration of MAT2A-associated genomic regions in bovine blastocysts using chromatin immunoprecipitation and sequencing (ChIP-seq) identified candidate MAT2A-associated genes implicated not only in short-term periconceptional embryo development, but also in long-term phenotypic programming during this period in terms of growth, metabolism, and immune functions. These results suggest the critical involvement of MAT2A in the periconceptional period in life-long programming of health and disease as well as successful preimplantation development.

## Introduction

The periconceptional period of mammalian embryo development is a critical window during which diverse environmental conditions, including available nutrients, not only have short-term effects on aspects such as cellular proliferation, but also long-term effects on metabolic and developmental processes throughout gestation and even during postnatal life^1^. In particular, preimplantation development is a period of dynamic epigenetic rearrangement involving substantial changes in DNA methylation and histone modifications, which may affect the regulation of specific and heritable patterns of gene expression^1, 2^.

Methionine adenosyltransferase (MAT, EC 2.5.1.6) is a family of enzymes that catalyses the synthesis of S-adenosylmethionine (SAM) from ATP and methionine^3^. MAT-mediated SAM synthesis is the first step in the methionine cycle, which plays a central role in transsulfuration, polyamine synthesis, and transmethylation pathways^4^. Due to its role as the immediate precursor of SAM, which is the universal methyl donor for epigenetic methylation of DNA and histones^5^, methionine, along with the other nutrients and metabolites that constitute one-carbon metabolism (OCM) such as folate, betaine, and vitamin B12, have received attention as nutritional factors that can influence the epigenetic regulation of gene expression^6, 7^.

Increasing evidence indicates that preimplantation development is vulnerable to disruptions in OCM^8–13^. For example, disruption of methionine metabolism by ethionine, an antimetabolite of methionine, impairs blastocyst development of mammalian preimplantation embryos *in vitro* ^9, 10, 13^. Analogously, inhibition of the folate cycle^11, 12^ and/or betaine-homocysteine methyltransferase knockdown^12^ also causes defective preimplantation development. In addition, the involvement of OCM in epigenetic modification of gene expression is also of interest, not only in terms of successful embryonic development, but also in relation to its possible association with nutrition-dependent phenotypic programming during the periconceptional period^14–16^.

Recently, mammalian oocytes and preimplantation embryos have been shown to express OCM-related enzymes, including MAT^8, 11, 12, 17^. In mammals, three *MAT* genes (*MAT1A*, *MAT2A*, and *MAT2B*) encode three MAT isozymes; the MATI and MATIII isozymes are a tetramer and dimer, respectively, of an identical catalytic subunit encoded by the *MAT1A* gene, whereas the MATII isozyme is a hetero-oligomer consisting of catalytic subunit(s) encoded by *MAT2A* and regulatory subunit(s) encoded by *MAT2B* ^3^. In our previous study, we found that bovine preimplantation embryos expressed *MAT1A* transcripts, which disappeared from the 8-cell stage onwards, with MAT1A protein decreasing from the morula stage, whereas *MAT2A* and *MAT2B* transcripts were expressed throughout the preimplantation development^8^. The results suggest that the MATII isozyme can function throughout preimplantation development. In the present study, we investigated the protein expression of MAT2A in bovine oocytes and preimplantation embryos and the roles of MAT2A in preimplantation development by using a MAT2A inhibitor, fluorinated N,N-dialkylaminostilbene-5 (FIDAS)^18^.

In the context of long-term phenotypic programming by OCM modulation during the periconceptional period^14–16^, possible interactions between MAT2A and the epigenetic status of specific genes are of particular interest. MAT2A has been reported to interact with many chromatin-related proteins and be recruited to their specific target genes to form gene-regulatory complexes^19–21^. Therefore, to address possible interactions of MAT2A with specific genomic DNA regions in the periconceptional period, we performed ChIP-seq analysis of bovine blastocysts using MAT2A antibody.

## Results

### MAT2A protein expression in bovine oocytes and preimplantation embryos

**Figure 1:**
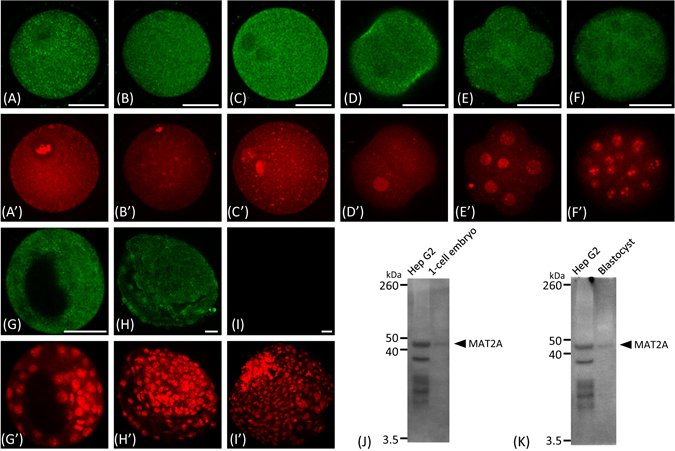
Immunofluorescence analysis of MAT2A protein in bovine oocytes and preimplantation embryos. Confocal images that transverse at least one nucleus or pronucleus are shown. (**A**) Immature oocyte, (**B**) mature oocyte, (**C**) 1-cell, (**D**) 2-cell, (**E**) 8-cell, (**F**) morula, (**G**) blastocyst, (**H**) hatched blastocyst, and (**I**) negative control in which the primary antibody was omitted from the immunofluorescence of a hatched blastocyst sample. Scale bars represent 50 µm. (**A’**–**I’**) Nuclear counterstaining with propidium iodide. (**J**) and (**K**) Immunoblotting of 1-cell embryo (n = 120) and blastocyst (n = 120) lysates using the monoclonal MAT2A antibody used in the immunofluorescence and ChIP-seq analyses. Hep G2 Cell Lysate (50 µg protein) was used as a positive control.

Immunofluorescence analysis revealed that MAT2A protein was expressed in bovine oocytes and preimplantation embryos throughout development. MAT2A was localized to both the nucleus and cytoplasm exclusively at equal levels or in a cytoplasm-dominant manner (Fig. 1A–H). At the blastocyst stage, some cells exhibited nuclear-dominant MAT2A localization (Fig. 1H and Fig. S1). Omission of the primary antibody resulted in no significant signal (Fig. 1I) and immunoblotting of 1-cell embryos and blastocyst lysates gave a single band at approximately 44 kDa corresponding to the predicted (both in human and bovine cases) and published^22, 23^ molecular weight of MAT2A. This band was identical to the main band for the positive control, a Hep G2 cell lysate (Fig. 1J and K). The extra bands seen in the Hep G2 cells that were not in the blastocysts suggest the possible advantage of the latter sample in identifying MAT2A-specific interactions. Collectively, these results support the validity of the antibody used. The same monoclonal antibody was used in the subsequent ChIP-seq analysis.

### Effects of MAT2A inhibitor on preimplantation development *in vitro*

**Figure 2:**
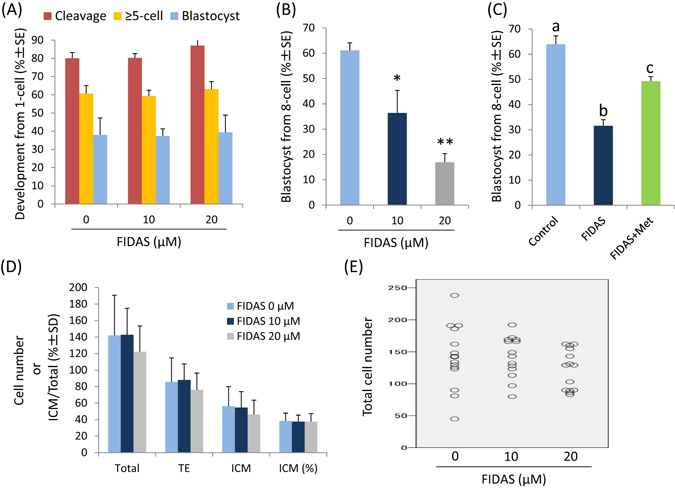
Effects of FIDAS on *in vitro* development of bovine embryos. (**A**) Effects of FIDAS during days 1 to 3 after fertilization. Cleavage and development to the ≥5-cell stage on day 3 and blastocyst development on day 8 are shown. Data were obtained from three to four replicates with 181, 182, and 138 embryos in the 0, 10, and 20 µM FIDAS groups, respectively. (**B**) Dose-dependent effects of FIDAS during days 3 to 8 after fertilization. Blastocyst development on day 8 from the 8-cell stage embryos is shown. Data were obtained from four replicates with 107, 106, and 107 embryos in the 0, 10, and 20 µM FIDAS groups, respectively. * and ** mean significant difference from the control at P < 0.05 and P < 0.01, respectively. (**C**) Effects of methionine (1 mM) on the FIDAS (10 µM)-induced impairment of blastocyst development. Data were obtained from four replicates with 110, 113, and 112 embryos in the control, FIDAS, and FIDAS + methionine groups, respectively. a, b, and c mean that values with different letters differ significantly (P < 0.01). (**D**) Effects of FIDAS during days 3 to 8 after fertilization on the total, ICM and TE cell numbers of blastocysts. Data were obtained from 15, 14, and 14 embryos in the 0, 10, and 20 µM FIDAS groups, respectively. (**E**) Distribution of the total cell number analysed in (**D**).

To determine the roles of MAT2A in preimplantation development, we examined the effects of FIDAS, a MAT2A inhibitor^18^, on the development of *in vitro*-produced bovine embryos. When FIDAS was added to the media during the first 52 h of *in vitro* culture, neither the rate of cleavage nor that of development to the ≥5-cell stage were affected at the end of the treatment. The subsequent development to the blastocyst stage after the removal of FIDAS was not affected either by the previous FIDAS treatment (Fig. 2A). However, the FIDAS treatment after 72 h post-insemination decreased (P < 0.01) the blastocyst development in a dose-dependent manner (Fig. 2B). The concomitant addition of excess methionine, the substrate of MAT2A and a competitor of FIDAS for MAT2A binding, significantly (P < 0.01) alleviated the FIDAS-induced reduction in blastocyst development (Fig. 2C). Although the mean number of blastocyst cells and their allocation to the inner cell mass (ICM) and trophectoderm (TE) were not affected by the FIDAS treatment during the ≥8-cell to blastocyst stage (Fig. 2D), the treatment narrowed the total cell number range (Fig. 2E).

### ChIP-seq analysis of bovine blastocyst-derived genomic DNA using MAT2A antibody

**Figure 3:**
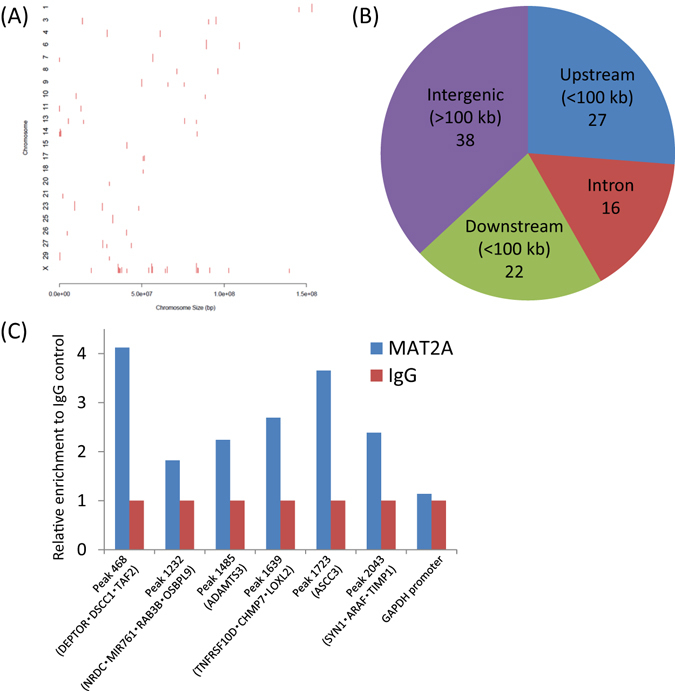
Characterization of MAT2A-ChIP specific peaks. (**A**) Chromosome distribution of 76 MAT2A-ChIP specific peaks. The figure was generated using the web-based CEAS (http://liulab.dfci.harvard.edu/CEAS/)^52^ tool. (**B**) Distribution of the peak-associated genes in terms of where the central point of the peak is located among different genomic regions. The bovine genome was divided into the following four categories: upstream (<100 kb) of a transcription start site (TSS), downstream (<100 kb) of a transcription end site (TES), the intron, and intergenic (>100 kb from a TSS or TES) regions. The numbers indicate those of genes for the upstream, intron, and downstream regions and those of peaks for the intergenic regions, respectively. (**C**) ChIP-qPCR validation of several MAT2A-associated peaks. Relative enrichments to IgG control are shown as the mean value of two to five ChIP-qPCR analyses for each peak. The peaks are shown with ID number (Table S1) and their associated genes in parentheses. The *GAPDH* promoter was used as a negative control for enrichment.

To address the possible interactions of MAT2A with specific genomic DNA regions^20, 21^ in bovine blastocysts, we performed ChIP-seq analysis using MAT2A antibody. ChIP-seq with Input, MAT2A antibody-ChIPed (MAT2A-ChIP), and control IgG-ChIPed (IgG control) DNA from duplicates furnished 2.5–2.9 × 10^7^ reads for each sample, 62–75% of which could be uniquely mapped to bosTau8 as a reference genome. Calculation of ChIP-enriched regions (peak calling) identified approximately 2,000 peaks from each sample (Table 1). We extracted 76 peaks in total that were specific for MAT2A-ChIP samples against either Input or IgG control throughout the duplicated ChIP-seq (Table S1, Fig. S2). The peaks were distributed to 21 autosomes and the X chromosome (Fig. 3A). The 76 peaks within the bovine genome were distributed into four kinds of regions—100 kb upstream of a transcription start site (TSS), the intron, 100 kb downstream of a transcription end site (TES), and intergenic regions^24^; 65 genes (Table S1) had one of these peaks within the intron or <100 kb upstream or downstream (Fig. 3B). These peaks and genes were designated gene-associated peaks and peak-associated genes, respectively.

**Table 1:** Data generated by ChIP-seq analysis of bovine blastocyst using MAT2A antibody.

Sample	Reads	Mapped reads* (%)	Called peaks
Input_1	28,691,648	21,639,966 (75.4)	2,023
MAT2A-ChIP_1	27,721,194	19,832,337 (71.5)	2,094
IgG control_1	29,394,375	19,837,199 (67.5)	1,935
Input_2	25,095,565	18,598,135 (74.1)	1,854
MAT2A-ChIP_2	25,583,060	17,176,033 (67.1)	1,788
IgG control_2	28,156,539	17,615,228 (62.6)	1,878

*Multi-mapped reads are not included.

**Figure 4:**
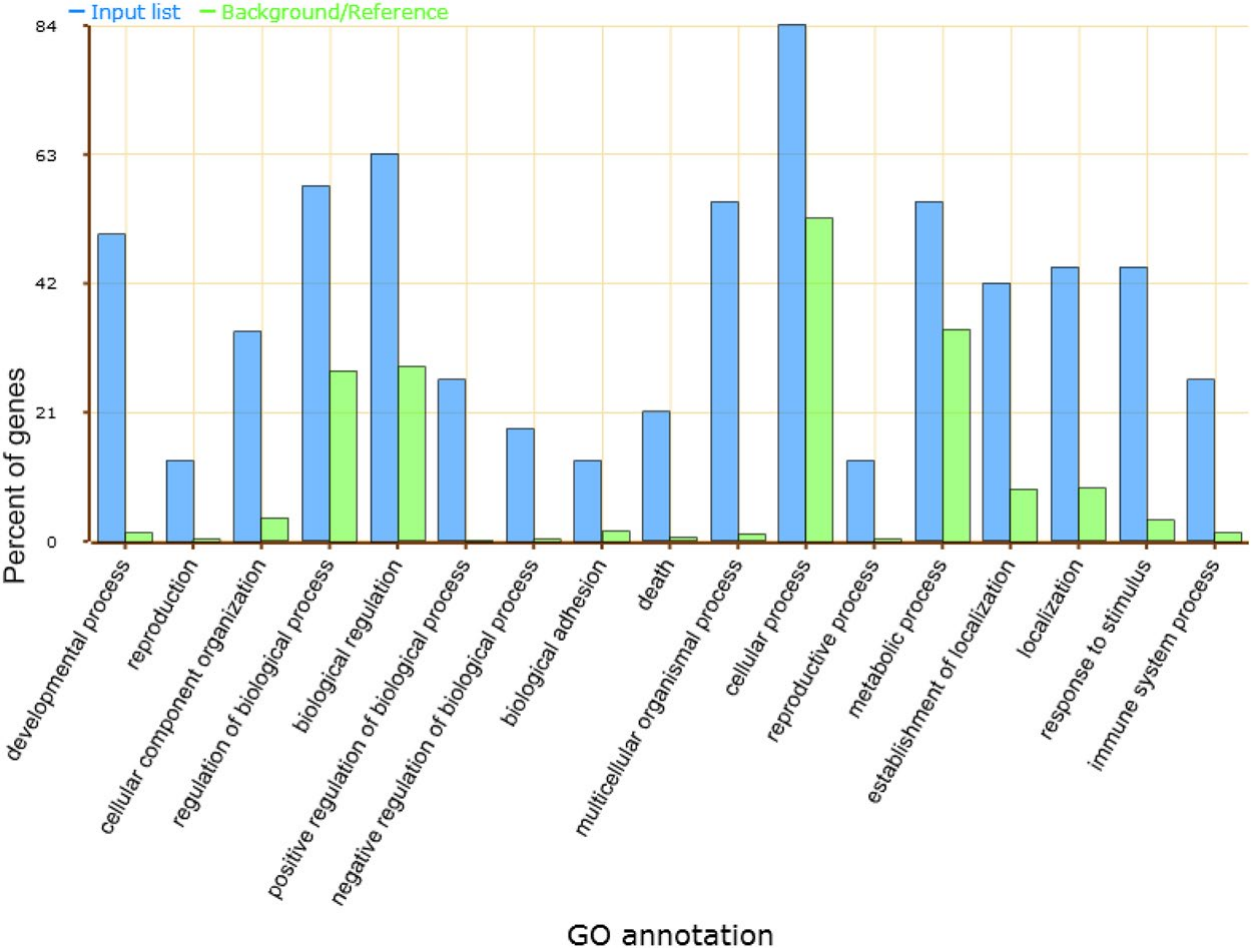
Gene ontology (GO) annotation in terms of biological process enriched from 35 genes associated with the MAT2A-ChIP specific peaks. All significant (P < 0.05) secondary level terms obtained as output from a web-based GO analysis toolkit, AgriGO^25^ (http://bioinfo.cau.edu.cn/agriGO/), are shown. Blue columns show the percentage of genes among the peak-associated genes, and green columns show the percentage of genes within the whole genome.

ChIP-qPCR validation of several gene-associated peaks confirmed their overall enrichment by MAT2A-antibody compared with the IgG control (Fig. 3C). Among the 65 peak-associated genes, 38 could be processed to gene ontology (GO) analysis using the web-based AgriGO^25^ tool. The analysis showed that biological process GO terms, such as ‘multicellular organismal process’, ‘developmental process’, and ‘positive regulation of biological process’, were enriched (Fig. 4, Fig. S3, Table 2, and Table S2).

**Table 2:** Significant biological process GO terms and constituent genes of particular interest to the study.

Accession	Description	Genes
GO:0048869	cellular developmental process	PPARD BEX5 TIMP1 RAB3B KIRREL3 MAEA B3GNT8 ELMO2 SPON2 PEAR1 HDAC8
GO:0030154	cell differentiation	
GO:0042981	regulation of apoptosis	BEX5 TNFRSF10D ARAF TIMP1 WRN
GO:0000003	reproduction	PPARD ELMO2 RAB3B HDAC8 DYSF
GO:0022414	reproductive process	
GO:0048856	anatomical structure development	PPARD BEX5 HDAC8 TIMP1 RAB3B KIRREL3 CXCR3 DEF6 ARAF MAEA B3GNT8 SPON2 PEAR1 DYSF
GO:0007275	multicellular organismal development	PPARD BEX5 ZNF280B TIMP1 RAB3B NXF3 KIRREL3 CXCR3 DEF6 ARAF MAEA B3GNT8 HDAC8 SPON2 PEAR1 DYSF WRN
GO:0008152	metabolic process	PPARD PIK3R4 LRRC71 ZNF280B LOXL2 AOAH ABCA1 RAB3B DEPTOR KIRREL3 DEF6 ARAF HDAC8 B3GNT8 ZNF674 PEAR1 BHMT OSBPL9 BCKDHA WRN TIMP1
GO:0006629	lipid metabolic process	PPARD AOAH ABCA1 BCKDHA OSBPL9
GO:0002376	immune system process	KIR2DL1 LRRC71 TIMP1 KIRREL3 CXCR3 DEF6 MAEA SPON2 VAMP7 HDAC8
GO:0006955	immune response	KIR2DL1 LRRC71 DEF6 SPON2 VAMP7 HDAC8

## Discussion

MATII is a ubiquitously expressed-type of MAT isozymes that catalyses the synthesis of SAM from ATP and methionine^3^. We first investigated the protein expression of the catalytic subunit of MATII, named MAT2A (also designated α2^3^), in bovine oocytes and preimplantation embryos up to the blastocyst stage, immunologically detecting MAT2A protein in all of these stages (Fig. 1A–I). This result is consistent with the previous finding that *MAT2A* mRNA is expressed throughout this period^8^. In contrast, the protein expression of the MAT1A (α1) subunit, which constitutes the isozymes of the adult liver dominant-type of MAT (MATI/III)^26^, reportedly decreases at the morula and blastocyst stages, following the disappearance of its mRNA from the 8-cell stage onwards^8^. Therefore, MATII is thought to be a dominant MAT, at least during the late stage of blastocyst development. Importantly, MAT2A immunofluorescence could be detected not only in the cytoplasm, but also in the nuclei of oocytes and embryos, suggesting the interaction of MAT2A with the genome during preimplantation development.

The addition of the MAT2A inhibitor FIDAS during the early cleavage stage (from the 1-cell to 8-cell stage) did not affect cleavage and the subsequent blastocyst formation after inhibitor removal (Fig. 2A). We previously reported that disruption of methionine metabolism by using ethionine, an antimetabolite of methionine, during the early cleavage stage also failed to affect development during this period^9^. Therefore, insensitivity to the MAT2A inhibitor during the early cleavage stage may be due to the dispensability of methionine metabolism in the early cleavage period rather than the possible compensating activity of the MAT1A gene products that exist during this period^8^. On the other hand, FIDAS treatment after the ≥8-cell stage dose-dependently decreased blastocyst development (Fig. 2B), and the inhibitory effect was significantly alleviated by the concomitant addition of a high concentration of methionine as both the substrate of MAT2A and the competitor of FIDAS for MAT2A binding (Fig. 2C). The FIDAS treatment also narrowed the blastocyst cell number range (Fig. 2D and E), suggesting that it affected both cellular proliferation and blastocoel formation. These results show that MAT2A function is essential for normal preimplantation development, particularly in terms of blastocyst development. In our previous studies, we showed the importance of methionine metabolism in blastocyst development of mammalian early embryos by using ethionine, a structural analogue of methionine that competitively inhibits many biological reactions involving methionine^9, 10^. The present results shed further light on the particular importance of SAM biosynthesis from methionine catalysed by MAT2A and provide novel evidence of the critical roles of OCM in mammalian preimplantation development accumulatively identified by several researchers, including our group^8–13, 27^.

MAT2A is recruited to specific genomic regions via interactions with other chromatin regulatory proteins in order to modulate the chromatin-remodelling of gene-regulatory elements of associated genes and consequently regulate their transcriptions^19–21^. In relation to the roles of MAT2A in blastocyst development revealed in the present study and in long-term phenotypic programming induced by the modulation of OCM during the periconceptional period^14–16^, we tried to explore the MAT2A-binding genomic regions in bovine blastocysts by ChIP-seq analysis using MAT2A antibody. We identified 76 MAT2A-ChIP specific peaks (Fig. 3A) and the interactions of some of these peaks with MAT2A were confirmed by ChIP-qPCR analyses, thereby validating the present ChIP-seq method (Fig. 3C).

The output of the GO analysis of the peak-associated genes identified many biological process GO terms (Fig. 4, Fig. S3, and Table S2) that could be related to biological phenomena of interest (Table 2). The genes *PPARD* and *TIMP1* contributed to the enrichment of the term ‘cellular developmental process’ and ‘cell differentiation’ as specialized terms of the secondary level term ‘developmental process’ or ‘cellular process’. *TIMP1* was also used to enrich the term ‘regulation of apoptosis’ as a specialized term of the secondary level term ‘death’. PPARD is a member of the peroxisome proliferator-activated receptors (PPARs), a group of the nuclear receptor superfamily of ligand-dependent transcription factors. *PPARD* is expressed in mammalian preimplantation embryos^28–30^ and is essential for cell proliferation in blastocyst development^30^. Apoptosis is observed in mammalian blastocysts both as a normal biological process in mammalian preimplantation development and as a sensitive process to the environment surrounding the embryos^31^. *TIMP1* (tissue inhibitor of metalloproteinase 1) is significantly expressed in mammalian preimplantation embryos^32–34^ and is an embryotrophic factor for bovine preimplantation embryos^35^. Furthermore, genes contributing to the aforementioned GO terms included those significantly expressed in bovine blastocysts, such as *MAEA*, *ELMO2*, *HDAC8*, and *ARAF* ^36^. Collectively, these genes may be related to the function of MAT2A in blastocyst development via the regulation of cellular development, differentiation, and apoptosis. In addition, the terms ‘reproduction’ and ‘reproductive process’ were also enriched by *PPARD*, *ELMO2*, *RAB3B*, *HDAC8*, and *DYSF* genes, suggesting the involvement of MAT2A in the reproductive process within and beyond the periconceptional period.

MAT contributes to OCM, whose modulation during the periconceptional period (direct or via maternal diets) can have long-lasting phenotypic effects on (1) pre- and post-natal growth with alterations of body composition, (2) metabolic processes (including glucose homeostasis, lipid metabolism, and cardiovascular functions), and (3) immune functions, and these phenotypes closely overlap with the symptoms of human metabolic syndrome or syndrome X^12, 37, 38^. In terms of growth and metabolic processes, GO terms such as ‘anatomical structure development’, ‘multicellular organismal development’, and ‘metabolic process’ (encompassing ‘lipid metabolic process’) were enriched by the ChIP peak-associated genes. In particular, *PPARD* was here again used for the enrichment of all these terms. *PPARD* expression is more tissue-ubiquitous and more abundant in skeletal muscle than that of other PPAR members (*PPARA* and *PPARG*)^39, 40^. The activation of PPARδ encoded by the *PPARD* gene is related to the alteration of body weight, fat deposition, glucose handling, and serum lipid concentrations and profiles by increasing fatty acid transport and oxidation and thermogenesis via activation of related gene expressions in both skeletal muscle and adipose tissue^39, 40^. PPARδ activation is also implicated in increased fatty acid transport and oxidation in cardiac muscle and regulation of arterial atherogenic processes via influence on macrophage cholesterol homeostasis and inflammatory signalling^39^. In addition, the genes recruited to enrich the ‘metabolic process’ included *ABCA1* (ATP-binding cassette sub-family A member 1), a major reverse cholesterol transporter^41^ and *DEPTOR* (DEP domain-containing mTOR-interacting protein) which interacts with mTOR signalling^42^, a critical nutrient-sensing and signal-integrating mechanism with effects on growth and metabolism^43^.

The term ‘immune system process’ and its child term ‘immune response’ were also significant with several genes associated with innate and adaptive immune responses including the functions of natural killer cell (*KIR2DL1* [killer cell immunoglobulin like receptor, two Ig domains and long cytoplasmic tail 1])^44^ and T cell-signalling (*DEF6* [differentially expressed in FDCP 6]^45^ and *VAMP7* [vesicle-associated membrane protein 7]^46^). The possible involvement of these genes may well represent the developmental and metabolic characteristics of the periconceptionally OCM-modulated animals described above.

In conclusion, the present study demonstrated the importance of MAT2A function in preimplantation development, particularly in blastocyst development. Furthermore, the exploration of MAT2A-associated genomic regions in the blastocysts has provided insight into possible interactions between MAT2A and specific genes that might be involved not only in short-term periconceptional embryo development, but also in long-term phenotypic programming during this period in terms of growth, metabolism, and immune functions. Further studies are needed to elucidate the precise interactions between MAT2A and the featured genes, including their epigenetic and transcriptional status and this may help us to optimize the periconceptional environment by improving maternal dietary conditions and/or *in vitro* culture systems for assisted reproductive technologies for life-long health promotion and disease prevention as well as successful preimplantation development.

## Methods

### Ethics statement

This study was carried out in accordance with the Regulation on Animal Experimentation at Kyoto University. The bovine ovaries used in the study were purchased from a commercial abattoir as by-products of meat processing and the frozen bull semen was donated from a local livestock breeding centre. Both facilities handled animals in strict compliance with the related laws, regulations, and guidelines. No animals were handled on university premises during this study.

### *In vitro* production of bovine embryos

Bovine oocytes were recovered from the ovaries of Japanese Black or Japanese Black × Holstein F1. Groups of 10 cumulus-enclosed oocytes (CEOs) were *in vitro* matured (IVM) for 22 h in 50-µl drops of Medium 199 with Earle’s salts (Life Technologies) supplemented with 5% (v/v) foetal calf serum (FCS) and 0.2 IU/ml follicular-stimulating hormone (Kyoritsu Seiyaku). Matured CEOs were subjected to *in vitro* fertilization (IVF) with frozen-thawed sperm from a Japanese Black bull as described elsewhere^9^. The day of IVF and the beginning of insemination were designated day 0 and 0 h post-insemination (hpi), respectively. At 20 hpi, the resulting 1-cell embryos were freed from cumulus cells and subsequently *in vitro* cultured (IVC) in 500 µl of *in vitro* culture medium (IVCM)^9^. All of the media were used under a layer of mineral oil unless otherwise noted. The cultures were performed at 38.5 °C under 5% CO_2_ in air (IVM and IVF) or under 5% CO_2_, 5% O_2_, and 90% N_2_ (IVC).

### Immunofluorescence analysis of MAT2A protein

Oocytes and preimplantation embryos at each developmental stage were fixed in 10% (v/v) formalin neutral buffer solution (Wako Pure Chemical) for 1 h, washed in PBS containing 0.05% (v/v) Tween 20 (PBST) for 1 h, and subsequently permeabilized with 0.5% (v/v) Triton X-100 in PBS for 1 h at room temperature. After being washed in PBST for 1 h, the samples were treated with blocking solution (PBST supplemented with 1% (w/v) bovine serum albumin) for 1 h at room temperature. A mouse monoclonal anti-MAT2A antibody (clone AT3A2; ab86424, Abcam) was diluted 250 times with blocking solution and co-incubated with samples for 12 h at 4 °C. After being washed with blocking solution for 1 h, samples were incubated for 5 h at 4 °C with 1000 times-diluted secondary antibody (A21202; Invitrogen). Further, the nuclei were counterstained with 10 μg/ml propidium iodide in PBST for 20 min. The samples were washed with PBST and mounted onto slides with Vectashield mounting medium (Vector Laboratories). The slides were examined under a laser-scanning confocal microscope (Olympus). The specificity of the antibody was validated by omission of the primary antibody from the immunofluorescence procedure and immunoblotting of 1-cell embryo and blastocyst (n = 120 for each stage) lysates with Hep G2 Cell Lysate (50 µg protein, sc-2227; Santa Cruz Biotechnology) as a positive control.

### Preparation of FIDAS

The MAT2A inhibitor FIDAS^18^ was purchased from Merck Millipore and dissolved in dimethyl sulfoxide (DMSO) at 20 mM and stored at −20 °C until further use. The stock was further diluted in DMSO as necessary and added to IVCM at 0.1% (v/v) to make 10 or 20 µM FIDAS. The control was supplemented with 0.1% (v/v) DMSO. The FIDAS- or DMSO-supplemented media were used without the coverage of mineral oil.

### Effects of MAT2A inhibitor on the *in vitro* development of bovine embryos

First, to investigate the effects of FIDAS during the early cleavage stage, the 1-cell embryos (approximately n = 45 per culture) at 20 hpi (day 1) obtained as described above were cultured for a subsequent 52 h in 500 µl of IVCM supplemented with 0 (control), 10, or 20 µM FIDAS. At the end of the treatment (72 hpi), the cleavage rate and that of development to the ≥5-cell stage were recorded and only the embryos that had developed to the ≥5-cell stage were transferred into 500 µl IVCM supplemented with 5% (v/v) FCS and further cultured up to 192 hpi (day 8). The rate of blastocyst development was evaluated at the end of the culture. The cultures were replicated three or four times.

Next, to investigate the effects of FIDAS during the period from the ≥8-cell stage to blastocyst development, the ≥8-cell stage embryos (approximately n = 27 per culture) at 72 hpi were further cultured up to 192 hpi in 500 µl of IVCM supplemented with 0 (control), 10, or 20 µM FIDAS. To confirm the specificity of the FIDAS effects, methionine, which competes with FIDAS agents for MAT2A binding^18^ and acts as the substrate of MAT2A^47^, was added at 1 mM. The rate of development to the blastocyst stage was compared at 192 hpi. The cultures were replicated four times. In addition, to assess blastocyst cell number and their allocation to ICM and TE, the blastocysts were differentially stained with CDX2 immunolabelling according to the previously described method^10^.

### ChIP-seq analysis of bovine blastocyst-derived genomic DNA using MAT2A antibody

ChIP for small cell numbers, µChIP^48^, was performed with a True MicroChIP kit (Diagenode) according to the manufacturer’s instructions with some modifications. Bovine blastocysts were *in vitro* produced as described above using supplementation of 5% (v/v) FCS in IVCM from 72 hpi to increase blastocyst yield. At 192 hpi, approximately 110 blastocysts per culture were collected and crosslinked for 10 min with 1% formaldehyde in PBS containing 20 mM Na-butyrate (PBS-NaBu) and then quenched with 125 mM glycine for 10 min. The blastocysts were washed with PBS-NaBu containing protease inhibitor cocktail (PBS-NaBu-PIC), transferred into a 1.5-ml tube with a small volume of PBS-NaBu-PIC, and stored at −80 °C until a total of approximately 670 blastocysts were obtained by replicating the procedure six times. Two pools (biological replicates) of the approximately 670 blastocysts were subjected to the subsequent ChIP procedures.

These blastocysts were lysed in Lysis Buffer and mixed with PBS-NaBu-PIC. The sample was sonicated to shear chromatin using a Bioruptor UCD-250 (Cosmo Bio) for 20 × 30 s with 30-s pauses in ice-water. The sample was centrifuged for 10 min at 14,000 × g and the supernatant (330 μl) was transferred to a new tube and mixed with 330 µl of ChIP Buffer. The 660-µl sample of sheared chromatin was divided into 20 µl as an ‘Input’ and two aliquots of 320 µl. The latter two aliquots were mixed with 5 µg of the aforementioned anti-MAT2A antibody (MAT2A-ChIP) or mouse IgG_2B_ isotype control (MAB004; R&D Systems) as a negative control (IgG control) and incubated for 16 h at 4 °C with rotation at 40 rpm. Protein A-coated magnetic beads (20 µl) were added to the sample, which was then rotated at 40 rpm for 2 h at 4 °C. The DNA was eluted from the immunoprecipitate, decrosslinked, and subsequently purified with MicroChIP DiaPure columns (Diagenode). The same DNA purification method was also applied to the Input sample and resulted in 10 µl each of DNA (Input, MAT2A-ChIP, and IgG control).

The DNA samples were processed to library preparation for next-generation sequencing by using a MicroPlex Library Preparation kit v2 (Diagenode) following the manufacturer’s instructions. After cluster generation using Illumina cBot (Illumina), sequencing was performed on a HiSeq2500 (Illumina) as 100 base (single read). Sequencing reads, which were filtered to remove adapter sequences using Illumina bcl2fastq2 (Illumina), were aligned to the bovine genome (Bos_taurus_UMD_3.1.1/bosTau8, Jun. 2014) except for scaffolds using Bowtie (version 1.1.2)^49^. The peaks were called in the MAT2A-ChIP samples using the Model-based Analysis of ChIP-Seq (MACS, version 1.4.2) (http://liulab.dfci.harvard.edu/MACS/)^50^ with default settings and filtered against the corresponding significant peaks found in the Input or IgG control samples (Fig. S2). The filtered peaks (n = 76) that were common to the two replicates were designated MAT2A-ChIP specific peaks. The ChIP-seq datasets have been deposited in the Gene Expression Omnibus (GEO) of the NCBI with accession number GSE85836.

### Validation of ChIP-seq results by ChIP-qPCR

Approximately 120–150 blastocysts were subjected to the aforementioned ChIP procedure to obtain 10 µl each of DNA (Input, MAT2A-ChIP, and IgG control). These samples were processed to pre-amplification by OmniPlex technology^51^ using the GenomePlex Single Cell Whole Genome Amplification Kit (Sigma) according to the manufacturer’s instructions. The pre-amplified DNA was purified with the GenElute PCR Clean-Up Kit (Sigma) in a 50-µl volume. The pre-amplified DNA (2 µl) was used as a template in quantitative PCR (qPCR) using the StepOnePlus Real-time PCR system (Life Technologies). The primer pairs used are listed in Table S3. The enrichment in the MAT2A-ChIP sample was calculated as the percentage of the Input (%Input) from Ct values and expressed as relative enrichment to the IgG control. The *GAPDH* promoter region was used as a negative control for enrichment. The experiments were repeated two to five times for each gene.

### Gene ontology analysis

A GO analysis of the peak-associated genes was performed by Singular Enrichment Analysis (SEA) of the web-based AgriGO^25^ (http://bioinfo.cau.edu.cn/agriGO/) tool with Fisher test and Hochberg (FDR) adjustment.

### Statistical analyses

The data on embryonic development and cell numbers were subjected to a general linear model in which treatment was taken as a fixed variable. When multiple comparisons were made, the Tukey-Kramer test was used. These data are presented as means ± standard error or standard deviation. All analyses were performed using SPSS (SPSS Inc.). Significance was accepted at P < 0.05.

## Electronic supplementary material

Supplementary Information

Table S1

Table S2
