# Supplementary material for: Role of methionine adenosyltransferase 2A in bovine preimplantation development and its associated genomic regions
Source: Sci Rep. 2017 Jun 19;7:3800. doi: 10.1038/s41598-017-04003-1 (PMC5476596; doi:10.1038/s41598-017-04003-1)
Supplement: Supplementary file 1 — Supplementary Information [file 41598_2017_4003_MOESM1_ESM.pdf]

Role of methionine adenosyltransferase 2A in bovine preimplantation development and its associated genomic regions

Shuntaro Ikeda[1], Ryouka Kawahara-Miki[2], Hisataka Iwata[3], Miki Sugimoto[1] and Shinichi Kume[1]

[1] Laboratory of Animal Physiology and Functional Anatomy, Graduate School of Agriculture, Kyoto University, Kyoto 606-8502, Japan
[2] NODAI Genome Research Center, Tokyo University of Agriculture, Tokyo 156-8502, Japan
[3] Department of Animal Sciences, Tokyo University of Agriculture, Kanagawa 243-0034, Japan

Supplementary figure legends

Figure S1. Nuclear localization of MAT2A in bovine blastocyst cells. (A) Immunofluorescence image of MAT2A protein in blastocyst cells. Nuclei showing more abundant MAT2A compared with the cytoplasm are indicated by arrowheads. (B) Nuclear counterstaining with propidium iodide. (C) Merged image of (A) and (B). Scale bar represents 50 μm.

Figure S2. Identification of MAT2A-ChIP specific peaks in duplicated ChIP-seq (Rep 1 and Rep 2). (A) 39 peaks (intersection) are specific for MAT2A-ChIP samples against Input in both replicates. (B) 59 peaks (intersection) are specific for MAT2A-ChIP samples against IgG control in both replicates. (C) The union of these peaks makes 76 MAT2A-ChIP specific peaks with 22 overlapping peaks.

Figure S3. Hierarchical tree graph of overrepresented GO terms in biological process generated by Singular Enrichment Analysis (SEA) of agriGO (http://bioinfo.cau.edu.cn/agriGO/). The first line in each significant box represents the GO term ID with the adjusted P-value in parentheses, followed by the lines indicating the term definition. In the last line of the significant box, the first pair of numerals represents the number of genes associated with the GO term and that of all genes in the query list and the second pair of numerals represents those in the *Bos taurus* database. The degree of colour saturation of the boxes is positively correlated with the enrichment level of the term: yellow, <0.05; orange, <5e-03 - <5e-09; and red, <5e-10. Solid, dashed, and dotted lines represent two, one and zero enriched terms at both ends connected by the line, respectively.

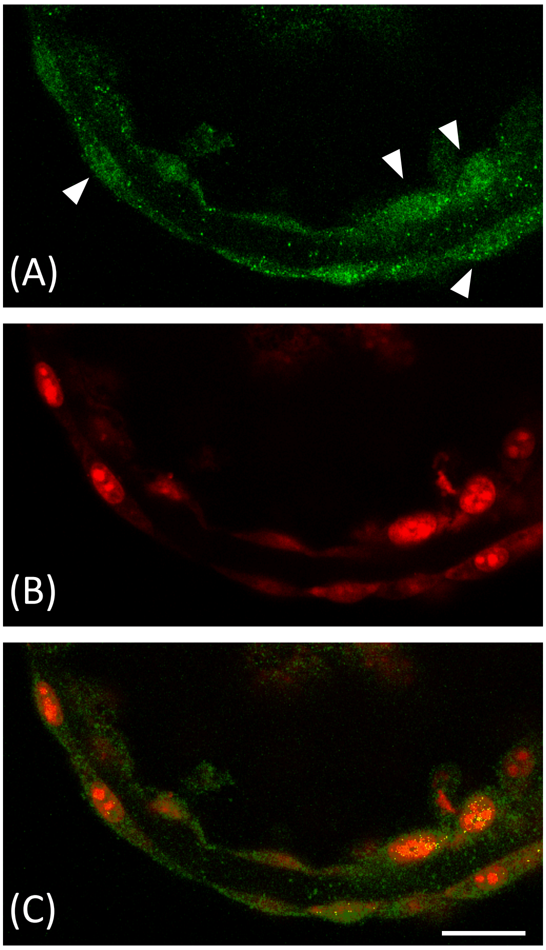

Figure S1

(A)

MAT2A-ChIP specific against

Input

(B)

MAT2A-ChIP specific against

IgG control

(C)

76 MAT2A-ChIP specific peaks

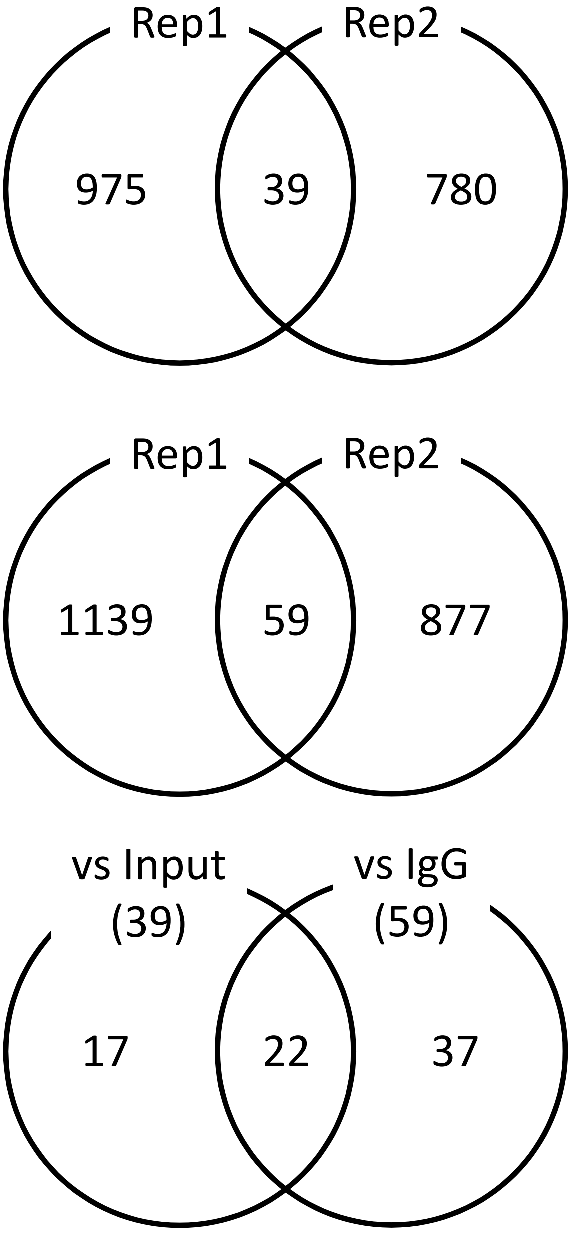

Figure S2

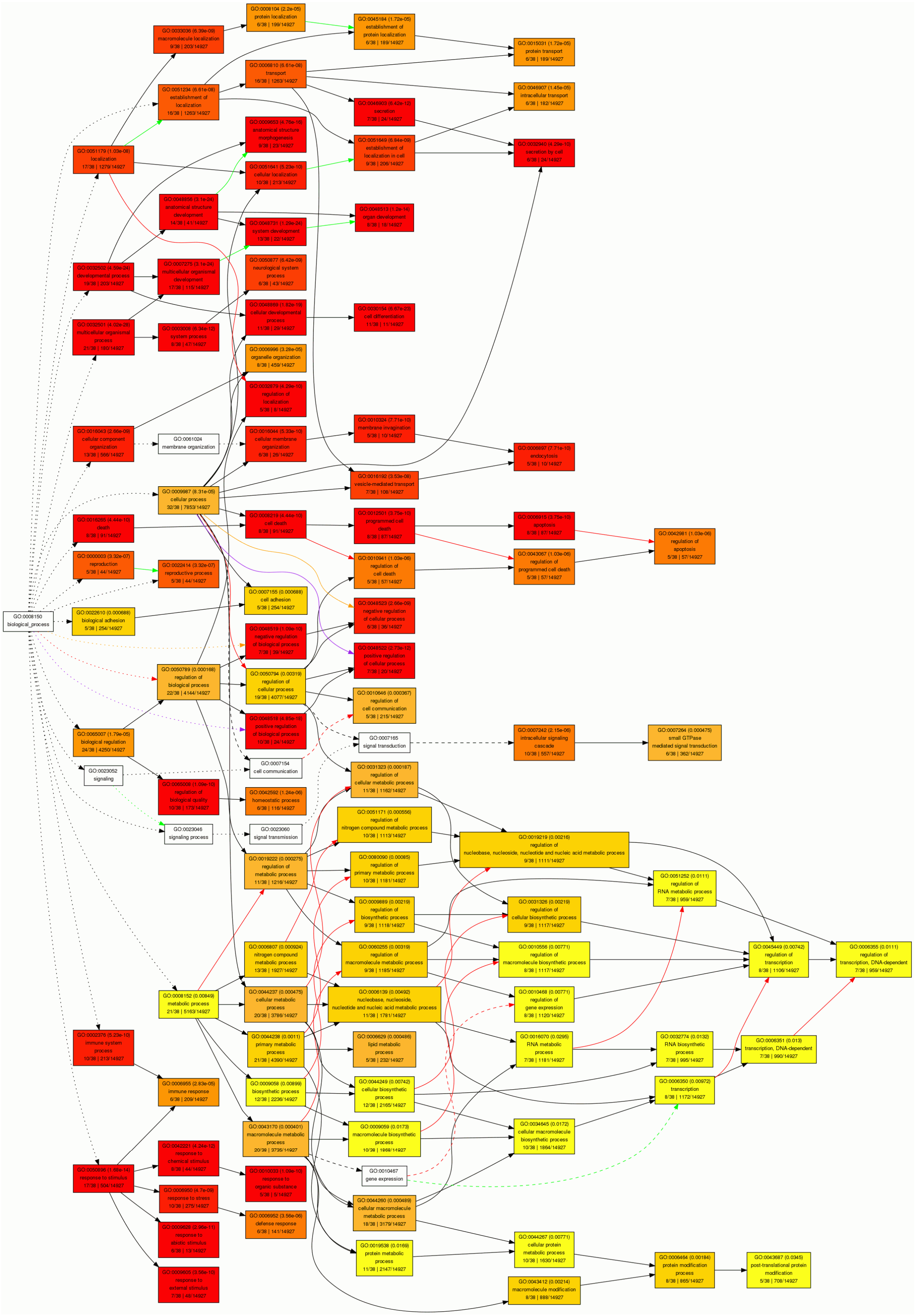

Figure S3

Table S3. The primer pairs used for validation of ChIP-seq peaks.

Peak ID	Primer sequences	
Peak 468	Forward	GGGTAGTTCCCCAAAGGCTG
	Reverse	CAAGGGCTGGGGCATGTAAT
Peak 1232	Forward	CAGCAGTCAGGCACCACTC
	Reverse	CTGCCCAGCTTCTAGCGT
Peak 1485	Forward	AAGTCAACACGAAGGGGCA
	Reverse	GGTTGAGGCATGGAACTCCG
Peak 1639	Forward	TTTGGTCTCTCTGTGCAGCTT
	Reverse	CCTGCTGTCTTATGGAGGGC
Peak 1723	Forward	TTCTGCCCTCTGACATGATTC
	Reverse	CAGATTGGACTGAAAGGACCA
Peak 2043	Forward	TAAGCACCTGGTGAACTGAG
	Reverse	TATTACGTGGCGTGTCAGG
GAPDH promoter *	Forward	TGTTATATCCTTGCGGCAGCTT
	Reverse	AGCACTGCGGGAGAGTAGTAAACT

* Herrmann D et al., Epigenetics 2013 8(3): 281–289 doi:10.4161/epi.23899
